# Gene Identification and Potential Drug Therapy for Drug-Resistant Melanoma with Bioinformatics and Deep Learning Technology

**DOI:** 10.1155/2022/2461055

**Published:** 2022-07-23

**Authors:** Muge Liu, Yingbin Xu

**Affiliations:** Department of Burn Surgery, The First Affiliated Hospital of Sun Yat-sen University, Guangzhou, China

## Abstract

**Background:**

Melanomas are skin malignant tumors that arise from melanocytes which are primarily treated with surgery, chemotherapy, targeted therapy, immunotherapy, radiation therapy, etc. Targeted therapy is a promising approach to treating advanced melanomas, but resistance always occurs. This study is aimed at identifying the potential target genes and candidate drugs for drug-resistant melanoma effectively with computational methods.

**Methods:**

Identification of genes associated with drug-resistant melanomas was conducted using the text mining tool pubmed2ensembl. Further gene screening was carried out by GO and KEGG pathway enrichment analyses. The PPI network was constructed using STRING database and Cytoscape. GEPIA was used to perform the survival analysis and conduct the Kaplan-Meier curve. Drugs targeted at these genes were selected in Pharmaprojects. The binding affinity scores of drug-target interactions were predicted by DeepPurpose.

**Results:**

A total of 433 genes were found associated with drug-resistant melanomas by text mining. The most statistically differential functional enriched pathways of GO and KEGG analyses contained 348 genes, and 27 hub genes were further screened out by MCODE in Cytoscape. Six genes were identified with statistical differences after survival analysis and literature review. 16 candidate drugs targeted at hub genes were found by Pharmaprojects under our restrictions. Finally, 11 ERBB2-targeted drugs with top affinity scores were predicted by DeepPurpose, including 10 ERBB2 kinase inhibitors and 1 antibody-drug conjugate.

**Conclusion:**

Text mining and bioinformatics are valuable methods for gene identification in drug discovery. DeepPurpose is an efficient and operative deep learning tool for predicting the DTI and selecting the candidate drugs.

## 1. Introduction

Melanoma is a severe skin malignant tumor that arises from melanocytes, which is the fifth most common malignant tumor in the United States. It accounts for the leading cause of skin cancer-related deaths [1]. The prognosis of melanoma is highly correlated with the pathology stage at first diagnosis—the patients with superficial melanoma (Breslow thickness ≤ 1 mm) have a higher cure rate [2]. Treatments of melanoma mainly include surgery, chemotherapy, targeted therapy, immunotherapy, oncolytic virus therapy, and radiation therapy [3]. Surgery is still an important method for melanoma. Wide excision is the classic surgical method for melanoma of the trunk and extremities [4–8]. Tumors growing in other body parts (head and neck, subungual, genitals, etc.) should be resected as thoroughly as possible and combined with postoperative reconstructive to improve appearance and function [9]. For advanced melanoma, resection of primary and metastatic tumors combined with adjuvant therapy (immunotherapy, targeted therapy, chemotherapy, etc.) has been proven to have a better prognosis [10–12].

As mentioned above, advanced melanomas require comprehensive therapies. Chemotherapy has not been shown to improve survival in patients with advanced melanomas [3, 13]. Radiation therapy is the palliative for local symptoms and consolidation for residual disease in patients who do not respond significantly to other systemic therapies. Checkpoint inhibitor immunotherapy and targeted therapy have made significant progress in recent years [14, 15].

Till now, researchers have accumulated many abnormally expressed genes in melanoma, and databases such as GEO and TCGA contain the sequencing data of melanoma specimens and clinical data of patients. We can screen out new critical genes affecting the survival of patients on this basis.

Besides the immunotherapy targets such as PD-1/PD-L1 and CTAL-4, the classic target genes in melanoma are BRAF- and MEK-related pathways. Mutation of BRAF (50%), NRAS (25%), and neurofibromin 1 (14%) are common in melanoma [16]. Small molecule inhibitors include Vemurafenib/Dabrafenib for BRAF and Trametinib/Cobimetinib for MEK.

However, resistance always occurs. It is currently believed that the targeted drug resistance mechanism of melanoma contains the following aspects: reactivation of the MAPK pathway [17], activation of substitutive pathways (PI3K-mTOR pathway) [18–21], alteration of the tumor microenvironment [22–24], autophagy and ER stress of tumor cells [25–27], miRNA-mediated resistance [28, 29], and therapy-mediated selection of resistant tumor cell subpopulations [30]. The mechanism of immune therapy resistance includes the immune desert, the immune-excluded tumor phenotype, increased regulatory cells in the tumor microenvironment, increased immunosuppressive cytokines, and upregulation of inhibitory receptors on T cells [31]. When targeted drugs or immune checkpoint inhibitors are ineffective, the current therapeutic schedule often combines drugs to achieve better survival than single agents, like MAPK pathway inhibitors and immune checkpoint drugs [32, 33]. However, the shortcomings are apparent. Drugs for melanoma are limited, and the effect of the drug combination is limited. If no new drugs are explored, there will inevitably be a situation where no more drugs are available.

Other targeted drugs have not been thoroughly studied and supported in the treatment of melanoma, such as the classic target gene-ERBB2 of breast cancer. Therefore, these targeted drugs provide new hope and convenience for exploring new treatment options for melanoma.

Traditional approaches to discovering a new drug are time-consuming and expensive, which can cause a substantial financial burden on society and delay in getting effective treatment for patients [34]. The task of finding a new drug is technologically tricky since the amount of drug-like molecules can be up to 10^60^ [35]. In the past decades, the emerging technology of computational methods is considered potential in the early stage of drug discovery [36]. Text mining is a technology based on massive data resources, allowing quick analysis of potential information [37]. It has been highly developed and successfully applied in fields like security applications, biomedical applications, and emotion analysis.

The application of artificial intelligence, particularly deep learning (DL), is acceleratingly impacting the field of biomedicine [38]. In drug research and development (R&D), the principal goal is to identify the compounds tightly and selectively to the target proteins, and DL is a powerful in silico tool in which many models are built for predicting the drug-target interaction (DTI) [39, 40]. Deep learning (DL) is prevailing in silico tool in biomedicine. Many DL models are built for predicting the drug-target interaction, compound property prediction, and protein-protein interaction prediction. DeepPurpose is a deep learning algorithm that provides a framework implementing over 50 advanced DL models, 15 drug encodings, and 8 target encodings based on many databases (BindingDB, DAVIS, KIBA, etc.). It is tested comparably effective to state-of-the-art DL models (GraphDTA and DeepDTA) [41]. DeepPurpose provides a simple framework to conduct DTI research using 8 encoders for drugs and 7 for proteins. The following steps correspond to one line of code in DeepPurpose.

- *Encoder Specification*. We select a specific encoder for drugs of SMILE format and proteins of amino acid sequence
- *Data Encoding and Split*. We use the selected encoders to convert the data into a format which can be recognized and calculated by DeepPurpose
- *Model Configuration Generation/Model Initialization*. Next, we adjust specific model parameters and initialize a model
- *Model Training*. We use the above data to train the model and output the results
- *Model Prediction and Repurposing/Screening*. After training the model, we use it to predict affinity scores on new data
- *Model Saving and Loading*. Finally, the model can be saved. The trained model can be saved and directly called for the prediction of new data [42]

In the present study, we identified the relevant genes of drug-resistant melanoma via text mining technology. Further, we screened the targeted genes with GO/KEGG/PPI/GEPIA analysis besides literature review. It was the first time that DeepPurpose was used to discover medicines for drug-resistant melanoma. It would provide a reference value in further study of the management of drug-resistant melanoma and other tumors.

## 2. Materials and Methods

### 2.1. Text Mining

Three phrases “melanoma,” “drug,” and “resistance” were input into pubmed2ensembl (http://pubmed2ensembl.ls.manchester.ac.uk/), a public source for mining the relevant biological literature on genes, which was used to obtain the associated gene list. We set “Homo sapiens” as the species and selected “Ensembl Gene ID,” “MEDLINE: PubMed ID,” and “Associated Gene Name.” “Search for PubMed IDs” and “filter on Entrez: PMID” were chosen for each query [43–46].

### 2.2. Biological Process and Pathway Analysis

Gene ontology (GO) and Kyoto Encyclopedia of Genes and Genomes (KEGG) pathway enrichment analyses were conducted by the Database for Annotation, Visualization and Integrated Discovery (DAVID) [47, 48], and the genes of the most statistically enriched pathways in GO and KEGG analyses (*p* less than 10*E*-20) were selected out and used for subsequent protein-protein interaction (PPI) analysis.

### 2.3. Protein-Protein Interaction

Protein-protein interaction (PPI) was conducted in Search Tools for the Retrieval of Interacting (STRING) database [49]. We imported candidate genes of the last step into the database and chose the “Homo sapiens” as the organism. Further, we imported the start and end nodes of STRING into Cytoscape to conduct the protein-protein interaction, using MCODE app to confirm the hub genes [50, 51].

### 2.4. Survival Analysis

The clinical significance of the candidate genes was validated by Gene Expression Profiling Interactive Analysis (GEPIA) [52]. The survival analysis results in GEPIA were used to screen out genes with significant statistical differences in skin cutaneous melanomas.

### 2.5. Drug-Gene Interaction

The pharmaprojects database (https://pharmaintelligence.informa.com) was used to inquire about drugs targeted at hub genes [53, 54]. Each hub genes generated drugs list targeted on it. Drugs with available SMILES structures, “launched,” “phase I/II/III clinical trial,” “pre-registration” or “registered” in global status, and “injectable” or “oral” in delivery routes were screened out for candidate drugs lists.

### 2.6. DeepPurpose

With target genes and their potential drugs, we employed DeepPurpose to calculate affinity scores between them [42]. 14 encoding combinations were chosen based on DAVIS, BindingDB, or KIBA. Affinity scores were calculated by importing SMILES structures of drugs and amino acid sequences of genes into pretrained models. We summarized the scores of each pair of drugs and target genes. Ultimately, we chose the drugs with affinity scores of at least 7.0 by DAVIS or BindingDB datasets and 12.1 by KIBA dataset [46].

### 2.7. Immunohistochemistry

We used the Human Protein Atlas to compare immunohistochemical staining of key genes between melanomas and skin melanocytes [55, 56].

## 3. Results

### 3.1. Identification of Targeted Genes

**Figure 1:**
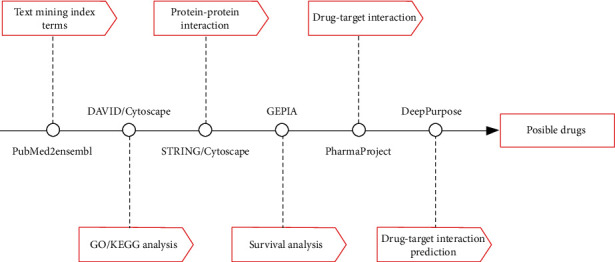
Flow chart of our study. Figure 1 shows the research process of our study. From left to right, the text labels represent the analysis contents and corresponding tools.

In the pubmed2ensembl, 433 genes related to “drug-resistant melanoma” were obtained after deleting duplicates. We carried out the text mining and exported related genes to excel on December 24, 2021. And the overall process is shown in Figure 1.

### 3.2. Enrichment Analysis of GO and KEGG of Associated Genes

**Figure 2:**
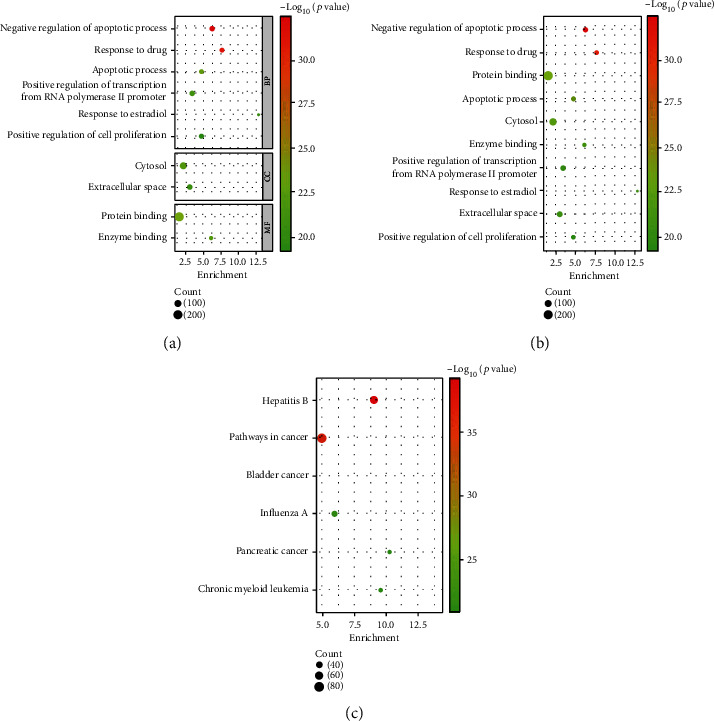
The most statistically significant processes/pathways in drug-resistant melanoma. (a) The biological process, cellular component, and molecular function of GO analysis in drug-resistant melanoma separately. (b) The biological process, cellular component, and molecular function of GO analysis in drug-resistant melanoma together. (c) The KEGG pathway analysis in drug-resistant melanoma. The size of the circles represents the count of genes, and the color represents the values of “- log10 (*p* value)”.

The most statistically differential processes in the GO and KEGG analyses were selected by *p* value (less than 10*E*-20) and shown in Figure 2. In GO analysis, “negative regulation of apoptotic process” (*p* = 3.84*E* − 33), “response to drug” (*p* = 1.94*E* − 31), “protein binding” (*p* = 2.45*E* − 24), “apoptotic process” (*p* = 2.59*E* − 24), “cytosol” (*p* = 3.31*E* − 23), “enzyme binding” (*p* = 4.95*E* − 23), “positive regulation of transcription from RNA polymerase II promoter” (*p* = 6.04*E* − 22), “response to estradiol” (*p* = 1.14*E* − 21), “extracellular space” (*p* = 4.95*E* − 21), and “positive regulation of cell proliferation” (*p* = 5.70*E* − 20) were the selected processes (Figures 2(a) and 2(b)). In KEGG analysis, “hepatitis B” (*p* = 7.40*E* − 40), “pathways in cancer” (*p* = 1.11*E* − 37), “bladder cancer” (*p* = 6.30*E* − 23), “influenza A” (*p* = 1.25*E* − 22), “pancreatic cancer” (*p* = 9.30*E* − 22), and “chronic myeloid leukemia” (*p* = 1.69*E* − 21) were screened out (Figure 2(c)). In total, 16 pathways and 348 genes were obtained.

### 3.3. PPI Network Analysis of Candidate Genes

**Figure 3:**
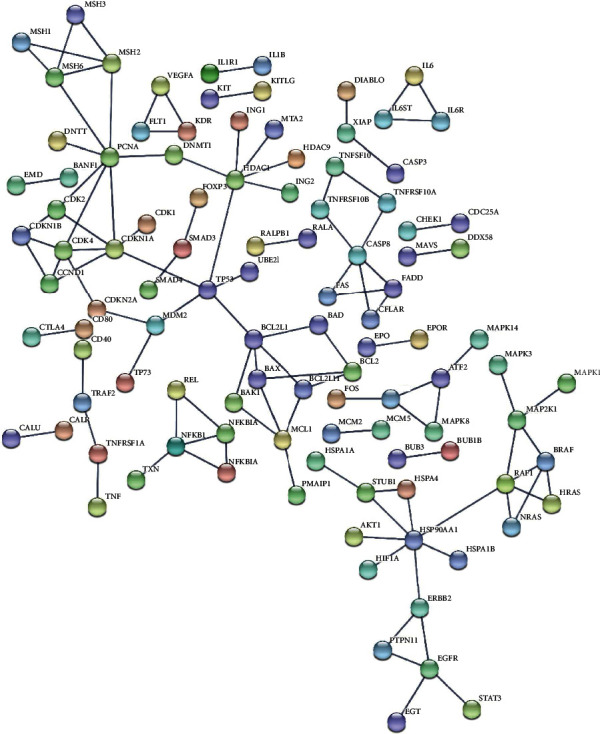
The protein-protein interaction of candidate genes. The protein-protein interaction analysis of candidate genes from STRING. Each circle represents one protein, and the line represents the interaction between them.

The PPI network created by Cytoscape is shown in Figure 3, and then, we imported the candidate genes into Cytoscape. After screening out by MCODE, 27 genes were obtained. Hub genes screened out by MCODE are shown in Table 1 and were chosen for further exploring.

### 3.4. Survival Analysis of Hub Genes

**Figure 4:**
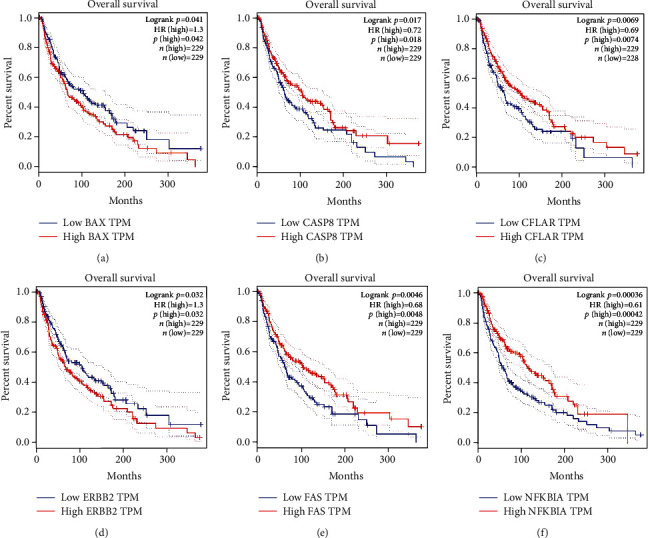
The survival analysis of hub genes in skin cutaneous melanomas from GEPIA. (a) The survival analysis of BAX in melanoma. Blue represents low BAX TPM, and red represents high BAX TPM. (b) The survival analysis of CASP8 in melanoma. Blue represents low CASP8 TPM, and red represents high CASP8 TPM. (c) The survival analysis of CFLAR in melanoma. Blue represents low CFLAR TPM, and red represents high CFLAR TPM. (d) The survival analysis of ERBB2 in melanoma. Blue represents low ERBB2 TPM, and red represents high ERBB2 TPM. (e) The survival analysis of FAS in melanoma. Blue represents low FAS TPM, and red represents high FAS TPM. (f) The survival analysis of NFKBIA in melanoma. Blue represents low NFKBIA TPM, and red represents high NFKBIA TPM.

The survival analysis of hub genes in skin cutaneous melanomas was conducted in GEPIA. Along with the article review, 6 genes including BAX (Figure 4(a)), CASP8 (Figure 4(b)), CFLAR (Figure 4(c)), ERBB2 (Figure 4(d)), FAS (Figure 4(e)), and NFKBIA (Figure 4(f)) with a statistical difference (*p* < 0.05) were screened out.

### 3.5. Targeted Drugs on Selected Genes in Pharmaprojects

**Figure 5:**
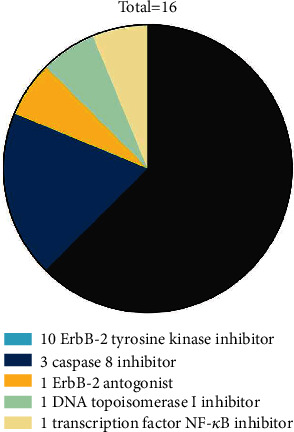
The candidate drugs for further DTI affinity score analysis. Each color represents a set of candidate drugs targeting the screened gene above. The area represents the proportion.

In Pharmaprojects, 16 drugs targeted on selected genes met the requirements (Figure 5), which included 10 ErbB-2 tyrosine kinase inhibitors, 3 Caspase 8 inhibitors, 1 ErbB-2 antagonist, 1 DNA topoisomerase I inhibitor, and 1 transcription factor NF-kappaB inhibitor.

### 3.6. Drug-Target Interaction Prediction by DeepPurpose

As shown in Table 2, the affinity scores calculated based on DAVIS and BindingDB datasets ranged from 3 to 9 approximately, while for KIBA dataset, the scores ranged from 10 to 13. As identifying the high-affinity drugs, the baseline score was set to 7.0 based on DAVIS or BindingDB, and 12.1 for KIBA. 11 drugs with further clinical verification values are screened out in Table 3. All of them were ERBB2-targeted drugs, including 10 ERBB2 kinase inhibitors and 1 antibody-drug conjugate.

### 3.7. The Protein Expression of ERBB2

**Figure 6:**
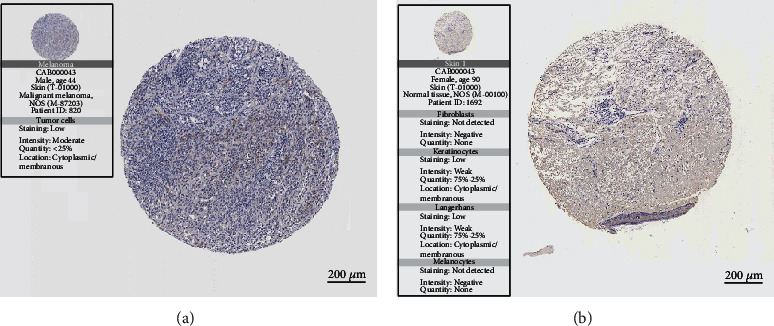
ERBB2 expression in melanomas and skin melanocytes. (a) The tissue specimen of melanoma with a moderate expression of ERBB2. (b) The tissue specimen of skin without an expression of ERBB2 in melanocytes.

After we identified ERBB2 as the promising target of drug-resistant melanoma by DeepPurpose. We used the Human Protein Atlas (HPA) database to confirm the protein expression in melanomas (Figure 6(a)) and skin melanocytes (Figure 6(b)). As shown in Figure 6, the protein expression of ERBB2 was higher in melanomas compared to the melanocytes in normal skin tissue. In melanoma, ERBB2 was detected by antibody CAB000043 with low staining, moderate-intensity and <25% quantity. While in skin melanocytes, ERBB2 was not detected by antibody CAB000043.

### 3.8. PPI Network Analysis of Six Hub Genes

**Figure 7:**
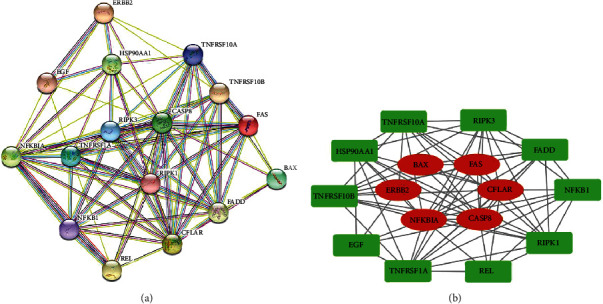
The protein-protein interaction of the six hub genes. (a) The protein-protein interaction analysis of the six hub genes from STRING. Each circle represents one protein, and the line represents the interaction between them. (b) The six hub proteins (red) and the closely related proteins interacting with them (green).

Finally, we constructed and analyzed the PPI relationship of six hub genes in Figure 7. CASP8 and NFKBIA were closely related to ERBB2, while BAX, FAS, and CFLAR were indirectly related to ERBB2. EGF, FADD, HSP90AA1, NFKB1, REL, RIPK1, RIPK3, TNFRSF10A, TNFRSF10B, and TNFRSF1A formed an interaction network with the six hub genes.

## 4. Discussion

This study purports to repurpose existing drugs as new drug options which have not been used for drug-resistant melanoma. Unlike previous methods of biomarker selection, our study did not focus on the mechanism of drug resistance but aimed at selecting the most potential gene targets through bioinformatics analysis. This study first obtained a wide range of candidate genes associated with drug resistance in melanoma (433 genes). Then, through GO and KEGG analysis enrichment, we selected genes in the most significant pathways for the next step (348 genes). Next, we screened 27 hub genes through PPI analysis and MCODE application in Cytoscape. In order to make it more clinically significant, survival analysis was conducted for candidate genes. Six genes with statistical significance were screened out for existing targeted drugs in Pharmprojects. Finally, we calculated the DTI and obtained the drugs with the highest affinity scores (11 drugs).

In the study, a total of 11 candidate compounds targeted on ERBB2 were identified. All of them were ERBB2-targeted drugs, including 10 ERBB2 kinase inhibitors and 1 antibody-drug conjugate.

The abnormal expression of the ERBB2 gene had been studied in melanoma. A study by Gottesdiener et al. included patients with nonuveal melanoma at Memorial Sloan Kettering Cancer Center from 2014 to 2018. In 732 melanoma cases, ERBB2 amplifications were detected in acral (3%) and mucosal (3%) melanomas. ERBB2 mutations were found in cutaneous (1%), acral (2%), and mucosal (2%) melanomas. ERBB2 amplifications were detected in acral (7%) and mucosal (6%) melanoma among 140 patients without canonical driver alterations. ERBB2 amplification was found in a patient resistant to checkpoint inhibition therapy, who showed a durable complete response to trastuzumab emtansine [57]. The research by Kluger et al. included 600 patients, and 31 patients had positive ERBB2 expression. 7% of patients had positive ERBB2 staining in primary cutaneous specimens, while 3.6% in recurrent or metastatic specimens. ERBB2 expression was associated with melanoma lesions with a Breslow depth of <2 mm [58]. In conclusion, abnormal expression of ERBB2 was associated with the development of melanoma and might be independent of the canonical driver. As a target, preliminary efficacy had been achieved in treating drug-resistant melanoma.

ERBB2 plays an important role in normal cell and tumor development. Erb-B2 Receptor Tyrosine Kinase 2 (ERBB2) is one of the epidermal growth factor receptor families. EGFR family contains four tyrosine kinase receptors: HER1, ERBB2, HER3, and HER4 [59]. The ligand-binding domain, transmembrane domain, and tyrosine kinase domain are the canonical structures of epidermal growth factor receptors. Till now, no endogenous ligands have been found for ERBB2. The ligand-independent manner or heterodimers with other EGFRs/tyrosine kinase superfamily can activate ERBB2 [60–62].

The ERBB2-PI3K-AKT signaling pathway is important for cell proliferation, protein synthesis, cell cycle progression, and survival [63]. AKT suppresses cell death and promotes cell survival in cancer cells [64]. AKT inhibits the expression of FKHR, FKHRL1, and AFX, which regulate apoptosis [63]. AKT also inhibits Bad, kinase ASK1, and procaspase9 [65–67]. Thus, AKT is essential for suppressing the induction of apoptosis. Mutations of AKT suppress cell proliferation, and upregulated AKT expression inhibits apoptosis [68, 69]. Besides, the PI3K-AKT-mTOR signaling pathway is a significant pathway regulating autophagy and tumorigenesis. For example, some tumor suppressor genes involved in TOR signaling (PTEN, TSC1, and TSC2) can stimulate autophagy [70].

The ERBB2-MAPK pathway is associated with cell proliferation, growth, and survival [71]. Activated ERK phosphorylates Bim to promote its ubiquitination, proteasomal degradation, and apoptosis [72, 73]. Study shows that ERBB2 causes apoptosis suppression by directly resulting in Puma destabilization and proteasomal degradation [74].

In our study, six hub genes were believed to be associated with drug resistance in melanoma. Among them, CASP8 and NFKBIA were closely related to ERBB2, while BAX, FAS, and CFLAR were indirectly related to ERBB2. After further expanding the PPI relationship, we found EGF, FADD, HSP90AA1, NFKB1, REL, RIPK1, RIPK3, TNFRSF10A, TNFRSF10B, and TNFRSF1A, as essential proteins, formed protein networks closely related to the above six hub genes. This interaction relationship can be the basis for subsequent studies on the mechanism of drug resistance in melanoma with ERBB2 as the entry point. The regulatory relationship between them can be further verified through experiments.

The anti-ERBB2 therapy contains three aspects: ERBB2-targeted monoclonal antibodies, antibody-drug conjugates, and ERBB2 kinase inhibitors. Monoclonal antibody drugs include trastuzumab and pertuzumab. Tyrosine kinase inhibitors include lapatinib, neratinib, pyrotinib, and tucatinib. Antibody-drug conjugates include trastuzumab emtansine (T-DM1) and Trastuzumab Deruxtecan (DS-8201). Our screened 11 drugs have a high affinity with ERBB2, which can play a good role in recognizing and blocking it. Among the 11 ERBB2-targeted drugs registered in the pharmaprojects database, the antibody-drug conjugates “Trastuzumab Deruxtecan” and ten other tyrosine kinase inhibitors were included.

None of the 11 drugs played a role in the treatment of melanomas. Mobocertinib is currently mainly used to treat lung cancer, while the remaining 9 tyrosine kinase inhibitors and Trastuzumab Deruxtecan have been used for various solid tumors, such as breast cancer, lung cancer, bladder cancer, kidney cancer, gastrointestinal cancer and nervous system malignancies.

As an essential factor in regulating cell death, ERBB2 plays a critical role in the occurrence of melanoma drug resistance. We screened out 11 drugs with the highest affinity for ERBB2 out of many existing drugs by deep learning algorithms. The treatment value for drug-resistant melanoma of these drugs deserves more exploration.

In conclusion, ERBB2 plays an essential role as a target in many tumors. Through machine learning, our study proves that ERBB2-targeted drugs may play an important role in treating drug-resistant melanoma. However, the research on the role of ERBB2 in melanoma is still insufficient, especially on the mechanism of drug resistance. We need more studies on the relationship between ERBB2 and melanoma resistance and developing it into medicine in the future.

## 5. Conclusion

In the present study, we explored the relevant genes of drug-resistant melanoma based on the technology of text mining. 433 genes were found by Pubmed2ensembl. Furthermore, the most statistically significant processes (*p* < 10*E* − 20) in the GO and KEGG analyses, respectively, were selected, and 348 genes were involved in these pathways. The PPI network was built in DAVIS and Cytoscape, where 27 genes were screened out by MCODE. Next, we got 6 genes with a statistical difference in survival analysis by GEPIA. For the implementation capability, the 16 targeted drugs were identified in Pharmaprojects under the stage of “launched” or “phase I/II/III clinical trial” or “pre-registration” or “registered.” We employed DeepPurpose, a deep learning algorithm, to calculate the affinity score, and 11 drugs were screened out, which were 10 ERBB2 kinase inhibitors and 1 antibody-drug conjugate. Our study provided a reference value in the drug discovery at the early stage. Nevertheless, the effectiveness requires further validation from lab work and clinical trials.

## Figures and Tables

**Table 1:** Hub genes selected by Cytoscape (MCODE).

Cluster	Score	Nodes	Edges	Hub genes
1	3.333	4	5	NFKBIA, IKBKB, REL, NFKB1
2	3.333	4	5	CFLAR, FADD, FAS, CASP8
3	3	3	3	IL6R, IL6, IL6ST
4	3	3	3	KDR, FLT1, VEGFA
5	3	3	3	JUN, ATF2, MAPK8
6	2.8	6	7	HSPA4, HSP90AA1, PTPN11, STUB1, ERBB2, EGFR
7	2.667	4	4	MCL1, BAX, BCL2, BCL2L11

**Table 2:** The total affinity scores of candidate drugs calculated based on DAVIS/BindingDB/KIBA.

Target gene	Generic drug name	CNN_CNN_DAVIS	CNN_CNN_BindingDB	Morgan_CNN_BindingDB	Morgan_CNN_DAVIS	MPNN_CNN_BindingDB	MPNN_CNN_KIBA	MPNN_CNN_DAVIS	Transformer_CNN_BindingDB	Daylight_AAC_DAVIS	Daylight_AAC_KIBA	Daylight_AAC_BindingDB	Morgan_AAC_BindingDB	Morgan_AAC_KIBA	Morgan_AAC_DAVIS
Caspase 8	Emricasan	5.06	5.82	4.15	5.06	5.47	10.86	5.49	3.97	6.37	11.72	5.19	5.00	10.70	5.21
CFLAR	2nd generation Idronoxil, Noxopharm	5.14	6.32	4.42	5.04	5.54	10.95	5.43	3.74	5.08	11.59	5.66	3.07	11.31	5.11
CFLAR	Idronoxil, Noxopharm	5.14	6.32	4.42	5.04	5.54	10.95	5.43	3.74	5.08	11.59	5.66	3.07	11.31	5.11
ERBB2	Afatinib	4.99	6.95	7.56	6.10	7.68	11.59	7.37	3.52	8.28	11.83	8.16	7.75	12.35	6.04
ERBB2	Dacomitinib	4.99	5.32	5.25	6.19	7.09	11.60	5.67	7.07	7.74	13.33	7.18	7.31	12.89	6.19
ERBB2	Epertinib	4.93	6.77	5.16	5.74	7.79	11.64	6.14	5.86	7.29	11.84	5.15	4.09	12.34	5.37
ERBB2	Lapatinib Ditosylate	4.98	7.80	7.49	5.47	7.55	11.57	7.31	7.47	7.73	11.82	8.07	7.66	12.24	5.13
ERBB2	Mobocertinib	4.94	5.88	7.23	6.40	6.12	11.92	5.30	6.77	5.24	11.13	5.48	6.81	11.86	6.50
ERBB2	Nelipepimut-S	4.97	5.96	6.20	5.09	6.81	10.65	6.66	3.52	4.99	11.43	4.69	4.61	10.61	5.01
ERBB2	Neratinib	4.98	7.31	8.65	7.67	8.39	11.46	7.71	7.94	8.12	11.23	8.09	7.55	11.70	7.95
ERBB2	Poziotinib	4.80	6.82	5.31	6.23	6.27	11.42	6.24	5.34	6.90	12.62	5.67	6.34	11.98	6.10
ERBB2	Pyrotinib Dimaleate	4.87	7.26	6.95	7.38	7.72	11.41	6.53	4.72	8.28	11.25	8.00	7.21	12.53	7.75
ERBB2	Tesevatinib	4.97	6.29	5.69	5.60	6.47	11.62	6.17	3.52	6.28	12.86	5.56	6.28	12.90	6.21
ERBB2	Trastuzumab Deruxtecan	4.94	6.36	7.90	5.05	6.44	11.87	5.31	6.38	5.14	11.73	6.93	7.32	11.23	5.11
ERBB2	Tucatinib	4.95	6.03	4.99	5.75	5.71	11.75	5.97	3.52	5.65	11.55	6.43	5.58	12.58	5.85
NFKBIA	Genistein, Humanetics	5.12	4.01	4.38	5.07	4.08	11.74	4.90	4.09	5.05	10.84	4.13	4.31	10.28	5.18

**Table 3:** The drugs with highest affinity scores.

Generic drug name	Target gene	MAX binding score	Model	Drug disease
Afatinib	Erb-B2 Receptor Tyrosine Kinase 2	12.35	Morgan_AAC_KIBA	Cancer (lung, head and neck, bladder, renal, sarcoma/neuroectodermal, sarcoma/rhabdomyo, brain, breast, colorectal, endometrial, gastrointestinal, ovarian, prostate); chronic obstructive pulmonary disease
Dacomitinib	Erb-B2 Receptor Tyrosine Kinase 2	13.33	Daylight_AAC_KIBA	Cancer (lung, non-small-cell, brain, head and neck)
Epertinib	Erb-B2 Receptor Tyrosine Kinase 2	12.34	Morgan_AAC_KIBA	Cancer (breast, biliary, colorectal, gastrointestinal, liver, lung, oesophageal, pancreatic, prostate, renal, vaginal)
Lapatinib Ditosylate	Erb-B2 Receptor Tyrosine Kinase 2	12.24	Morgan_AAC_KIBA	Cancer (breast, gastro-oesophageal junction, bladder, brain, cervical, colorectal, gastrointestinal, head and neck, lung, ovarian, peritoneal, renal)
Mobocertinib	Erb-B2 Receptor Tyrosine Kinase 2	7.23	Morgan_CNN_BindingDB	Cancer (lung)
Neratinib	Erb-B2 Receptor Tyrosine Kinase 2	8.65	Morgan_CNN_BindingDB	Cancer (breast, biliary, bladder, cervical, colorectal, head and neck, lung, endometrial, gastrointestinal, ovarian)
Poziotinib	Erb-B2 Receptor Tyrosine Kinase 2	12.62	Daylight_AAC_KIBA	Cancer (lung, breast, colorectal, gastrointestinal, head and neck, oesophageal, pancreatic)
Pyrotinib Dimaleate	Erb-B2 Receptor Tyrosine Kinase 2	12.53	Morgan_AAC_KIBA	Cancer (breast, gastrointestinal, lung, biliary)
Tesevatinib	Erb-B2 Receptor Tyrosine Kinase 2	12.90	Morgan_AAC_KIBA	Cancer (adrenal, brain, breast, chordoma, colorectal, head and neck, lung, mesothelioma, ovarian); polycystic kidney disease
Trastuzumab Deruxtecan	Erb-B2 Receptor Tyrosine Kinase 2	7.90	Morgan_CNN_BindingDB	Cancer (breast, gastro-oesophageal junction, gastrointestinal, lung, biliary, bladder, cervical, colorectal, endometrial, ovarian, pancreatic, oesophageal)
Tucatinib	Erb-B2 Receptor Tyrosine Kinase 2	12.58	Morgan_AAC_KIBA	Cancer (breast, gastro-oesophageal junction, gastrointestinal, biliary, bladder, cervical, colorectal, endometrial, lung)

## Data Availability

The datasets used during the current study are available from the corresponding author on reasonable request.

## Abbreviations

AFX: Forkhead box o4
ASK1: Mitogen-activated protein kinase kinase kinase 5
Bad: BCL2-associated agonist of cell death
BAX: BCL2-associated X
BRAF: B-Raf protooncogene
CASP8: Caspase 8
CFLAR: CASP8 and FADD-like apoptosis regulator
DAVID: Database for Annotation, Visualization and Integrated Discovery
DL: Deep learning
DNA: Deoxyribonucleic acid
DTI: Drug-target interaction
EGF: Epidermal growth factor
EGFR: Epidermal growth factor receptor
ER: Endoplasmic reticulum
ERBB2: Erb-B2 Receptor Tyrosine Kinase 2
ERK: Mitogen-activated protein kinase 1
FADD: Fas-associated via death domain
FAS: Fas cell surface death receptor
FKHR: Forkhead box o1
FKHRL1: Forkhead box o3
GEPIA: Gene Expression Profiling Interactive Analysis
GO: Gene ontology
HER: Human epidermal growth factor receptor
HPA: Human Protein Atlas
HSP90AA1: Heat shock protein 90 alpha family class A member 1
KEGG: Kyoto Encyclopedia of Genes and Genomes
MAPK: Mitogen-activated protein kinase
MCODE: Molecular complex detection
MEK: Mitogen-activated protein kinase kinase
miRNA: Microribonucleic acid
mTOR: Mammalian target of rapamycin
NF1: Neurofibromin 1
NFKB1: Nuclear factor kappa B subunit 1
NFKBIA: NFKB inhibitor alpha
NRAS: NRAS protooncogene
PI3K: Phosphatidylinositol-3-kinase
PPI: Protein-protein interaction
Puma: BCL2 binding component 3
REL: REL protooncogene, NF-*κ*B subunit
RIPK1: Receptor interacting serine/threonine kinase 1
RIPK3: Receptor interacting serine/threonine kinase 3
SMILES: Simplified molecular-input line-entry system
STRING: Search Tools for the Retrieval of Interacting
TNFRSF10A: TNF receptor superfamily member 10a
TNFRSF10B: TNF receptor superfamily member 10b
TNFRSF1A: TNF receptor superfamily member 1A.
